# Smart System for Bicarbonate Control in Irrigation for Hydroponic Precision Farming

**DOI:** 10.3390/s18051333

**Published:** 2018-04-25

**Authors:** Carlos Cambra, Sandra Sendra, Jaime Lloret, Raquel Lacuesta

**Affiliations:** 1 Instituto de Investigación para la Gestión Integrada de zonas Costeras, Universitat Politècnica de València, 46730 Valencia, Spain; carcamb1@doctor.upv.es (C.C.); jlloret@dcom.upv.es (J.L.); 2 Department of Signal Theory, Telematics and Communications, Universidad de Granada, 18071 Granada, Spain; 3 Department of Computer Science and Engineering, Universidad de Zaragoza, 50018 Zaragoza, Spain; raquellacuesta@gmail.com

**Keywords:** wireless sensor networks (WSNs), Internet of Things (IoT), hydroponic agriculture, potential of hydrogen (pH) sensor, smart farming, precision agriculture

## Abstract

Improving the sustainability in agriculture is nowadays an important challenge. The automation of irrigation processes via low-cost sensors can to spread technological advances in a sector very influenced by economical costs. This article presents an auto-calibrated pH sensor able to detect and adjust the imbalances in the pH levels of the nutrient solution used in hydroponic agriculture. The sensor is composed by a pH probe and a set of micropumps that sequentially pour the different liquid solutions to maintain the sensor calibration and the water samples from the channels that contain the nutrient solution. To implement our architecture, we use an auto-calibrated pH sensor connected to a wireless node. Several nodes compose our wireless sensor networks (WSN) to control our greenhouse. The sensors periodically measure the pH level of each hydroponic support and send the information to a data base (DB) which stores and analyzes the data to warn farmers about the measures. The data can then be accessed through a user-friendly, web-based interface that can be accessed through the Internet by using desktop or mobile devices. This paper also shows the design and test bench for both the auto-calibrated pH sensor and the wireless network to check their correct operation.

## 1. Introduction

Over the last decades, traditional horticulture and greenhouse-growing have experienced an important evolution due to the advent of more environmentally friendly alternatives. Dry season periods and climate change generate a low storage nutrient solution quantity [1]. For this reason, it necessary to implement new strategies to treated wastewater to allow its reuse in irrigation. Thus, integrated horticulture requires lower water quality and irrigation systems involving advanced technology that is applied according to a bio-dynamic schedule based on water quality [2]. The latest revolutions in greenhouse-growing have been due to advances in technology and wireless communication [3]. Such technology is used in precision farming whereby large amounts of data can be analysed following collection by a Wireless Sensor Network (WSN). Measurements show significant spatial variability in temperature (T^a^) and humidity allowing the production of high volumes of a crop with the levels of quality required by the market. Precision farming helps farmers to monitor [4] and control the micro-climatic conditions in the greenhouse [5] and the diseases and pests that affect the plants. There are different environmental factors and micro-indoor climatic events that can affect the state of the plants. Temperature and relative humidity (RH) influence the development of plants (i.e., their growth and quick maturation) and the proliferation of some diseases. Drip irrigation can negatively impact the development of diseases derived from salinity, pH and fertilizer levels. An auto-compensated framework is focused on the prevention of false alkalinity data acquisition and blind drippers—a disease caused by irrigation and fertilizer optimization. It is one of the most damaging greenhouse production diseases in terms of greenhouse efficiency. When environmental and micro-indoor climatic conditions are favorable, it is easier to control the irrigation of water quality. It is important to note that when growing greenhouse crops, the point in the life cycle of the production at which particular nutrient solutions should be applied should be known and, it is critical to apply the right fertilizer treatments or drip system cleaner at the right time and at the right place. Despite the horticulture industry’s reputation of being environmentally safe, prior research has shown that cultivation and production of vegetables in greenhouses are associated with a large number of environmental issues [6], in some cases due to chemical treatments. Some of the main factors involved include water waste and its quality, the production and management of organic and inorganic solid waste, energy used to feed the entire system, the generation of Greenhouse Gas (GHG) emissions [7], the use and management of chemicals in the greenhouses, land use issues, and the impact on ecosystems.

Research [8] describing improvements in crops has generated growth in the area of greenhouse crops by around 10.5% in the last four years. This gradual increase in the greenhouse area in recent years has been reflected in the volume of fruit and vegetable production which has grown in by 20% over the last few seasons. In the 2010/2011 season, Almeria province (Spain) harvested 2.87 million tons; in 2011–2012, it produced just over three million tons; in 2012/2013, Almeria collected 3.13 million tons and in 2013/2014, according to the data provided by the government delegation, this Spanish province generated just over 3.4 million tons. These data show us how agriculture methods are changing to increase the quantity and quality of products. Therefore, we need to find new mechanisms to optimize production to cope with this increase.

This paper presents the design, implementation and testing of a smart system for bicarbonate control in irrigation for hydroponic precision farming that may help to improve the water quality in hydroponic agriculture. In addition, the process of calibration and measurement is controlled by a middleware (developed in Java) focused on reducing the quantity of false data acquired. The data is stored in a database (DB) that provides farmers with a customized auto-plan to control particular diseases, leading to a more sustainable agriculture. The system is also characterized by low operating and managing costs due to the use of auto-cleaner probes, an auto-calibration pH test (with buffer pH samples of 4, 7 and 10) and an auto-acid injection dosage of the nutrient solution. In addition, the monitoring system provides historical and real-time values of different relevant environmental parameters, generating statistics that may help farmers to make specific measures and decisions to improve fertilizer treatments and water quality. This system is part of the multimedia platform, PLATEM PA (Multimedia Technology Platform for precision agriculture) [9]. PLATEM PA has been designed to create a user interface based on intelligent decision rules in which different professionals from the agricultural sector can interact with the purpose of solving problems immediately and remotely. PLATEM PA allows user-intelligent system interaction (irrigation, greenhouse, actuators, etc.) and generates a place where the platform users can exchange opinions and experiences.

The usefulness of our technological solution can be shown by the following example involving a farmer working on the indoor production of tomatoes for a small horticulture cooperative. He controls the greenhouse parameters daily. He has at his disposal a historical dataset that describes the weekly recommended water times, water pH levels, and water electrical conductivity (EC) levels for tomato production over a one-year period. These recommendations have been determined with a rule-learning algorithm based on a classification model consisting of 65 rules that specify the levels of bicarbonates, indoor ultraviolet (UV) light and temperature, water time, fertilization and pest control.

Following the use of these rules, this farmer was not satisfied because of poor results. He deletes some rules which seem to be artifacts of the data and introduces new rules that represent well-known relationships between acidity levels and watering time that were omitted by the learning algorithm. After verifying the increase in accuracy of the edited rule set with a holdout dataset, he deploys the model. If the farmer had to rely on commodity rule learning systems, he probably would have had to export the rule learning results via XML, and then import the results to a scoring engine. The system presented in this paper allows this task to be accomplished through a web-based platform, the PLATEM multimedia platform, where the farmer can share his results with the rest of the cooperative farmers’ platform group.

The main reason for focusing our work on hydroponic agriculture [10] is because nowadays, it has been demonstrated that hydroponic agriculture is economically more sustainable than traditional agriculture [11]. It also allows greater growth in terms of height, that is, for the same area, we can develop vertical structures [12] with different supports. Finally, hydroponic agriculture is becoming the best option [11,13] for cultivation in countries harmed by the presence of uncultivable and scarce food growing areas.

The rest of paper is organized as follows: Section 2 describes the state of the art latest technologies applied to monitoring and data acquisition using WSN. Section 3 introduces models for preventing false data acquisition and the generation of good quality nutrient solution samples. Section 4 details the hardware and software components in system architecture. Section 5 describes the experimental setup and results of different tests performed in the smart irrigation framework inside PLATEM. Finally, Section 6 details the conclusions and future work.

## 2. Related Work

Over the last few years, different researchers and companies have proposed solutions for monitoring the state of crops (fields and greenhouses) by using sensors. Such sensors, when deployed throughout an area of crops, allow us to distinguish the specific necessities of each area which improves the management of the field and addresses possible problems accurately. This section shows some of the most important papers related to hydroponic agriculture. The section is organized as follows: First, the section presents the latest contributions in smart greenhouse control systems for hydroponic agriculture. After that, we include some works related to the use of WSN and sensor communication in agriculture. Finally, this section discusses platforms where farmers can share data regarding to farming and crops and how to add a smart core to multimedia platforms. We also describe the advantages of our system compared to the aforementioned literature.

In [14], the authors presented the water quality aspects of hydroponic cultivation for cases requiring excellent water quality. If the supply water contains certain elements, there are restrictions in its use. In the study, is recommended to always analyze the supply water before starting cultivation and during production. Another important work presented by R. Soranz [15] compared the performance of an irrigation system using self-compensating troughs filled with a nutrient film technique (NFT) hydroponic system for greenhouse lettuce production. The daily monitoring of EC and pH allow experimental management based on the recommended values for optimal lettuce growth. The importance of water quality in hydroponics is important not only for plants, but also for system functionality. In [16], the authors highlighted that water is one of the most critical inputs in nursery and greenhouse crop production, and periodic analysis of water quality composition is important. In the literature, different techniques for monitoring the health of crops and pest decision support are described [17], for example, a hydroponic monitoring and automation system allows users to monitor and control NFT hydroponic farming using a web interface management system that includes a responsive web framework. In [18], the authors described the development of an embedded, portable analyzer, incorporated with a sensor array of polyvinyl chloride (PVC) membrane-based, ion-selective electrodes (ISEs) to directly measure the concentrations of NO_3_, K, and H ions in hydroponic solutions. WSNs based on Wi-Fi standards (IEEE 802.11 a/b/g/n), Bluetooth and ZigBee have extensively been used in different fields for monitoring different parameters [19], appliances [20] and for coding equipment communication [21], in addition to other uses. An example of a WSN-based monitoring system that uses IEEE 802.11 a/b/g/n transceivers was presented in [22]. The system monitors parameters such as temperature and soil moisture and calculates irrigation based on field data and rule-based knowledge to make the best decisions. WSN methods based on ZigBee are widely used in different fields for monitoring different parameters [23]. Another ZigBee-based solution was detailed in [24]. ZigBee technology offers a long battery life, small size, high reliability, automatic or semi-automatic installation, and, in particular, low system cost. Therefore, it is a better choice for greenhouse monitoring and control than other wireless protocols.

There has been previous research ocused in the field of precision agriculture in which the development of sensors and the remote management of irrigation programs has been presented. A previous article about the H2020 Smart-Akis project presented knowledge about the contributions of European Farming Technologies and commercial technologies [25]. Additionally, reference [26] demonstrated that the use of Association Rule Classification algorithms, such as Classification Based on Associations algorithm (CBA) [27], solves the excessive number of rules contained within even small datasets, and allows contradicting rules to be generated without adversely impacting the quality of the classifier. A similar tool focused on agriculture was presented in [28] where the author proposed a cloud-based autonomic information system for delivering Agriculture-as-a-Service (AaaS) through the use of cloud and big data technology. The system gathers information from various users through preconfigured devices and IoT sensors and processes it in the cloud using big data analytics, subsequently providing the required information to users automatically. 

As the related literature has shown, there are several systems that are currently applied in hydroponic agriculture, including devices to control the pH, acidity or nutrient auto-dosage. However, all of them are applied in to water source without taking into account the current state of water that is already in the gutters that provide the nutrient solution to the plants. Our system measures the current acidity level in the gutters and adjusts the value of the new nutrient solution that will be provided to the plants to maintain an optimum acidity value at all times. This task is not performed by existing systems. In addition, we use a WSN to gather data from several points in the facility and verify that the nutrient solution throughout the entire facility is the adequate for its crops.

Our smart system presents several advantages with respect to existing solutions. First, unlike other systems, it provides a complete system, both hardware and software, so compatibility issues are minimized. Furthermore, this kind of development avoids the collection of data from or through third-party (and usually expensive) cloud-based platforms. Second, it uses non-proprietary communication technology (NRF24L01), which eases the integration of both nodes and third-party gathering data with auto-recalibrated sensors. Third, thanks to the use of standard NRF24L01 transceivers [29], the deployment cost of the system is low, while providing a good coverage area. Finally, our system provides a multimedia responsive platform and makes the task of monitoring data simple as it can be done via mobile devices or computers.

## 3. The Proposed System

This section describes the proposed system and its operation. The network architecture is also presented as well as the devices used to implement the nodes.

### 3.1. Sensor Description

The acidity of a nutrient solution is measured with a pH sensor. The pH level of the nutrient solution in this study requires a mildly acidic nutrient solution with a pH level ranging from 5.5 to 5.7 for optimum neutralization of bicarbonates and salts. The total pH scale ranges from 4.5 to 14, where 7.0 is considered the neutral pH value. A liquid solution with a pH lower than 7 is considered to be an acidic solution, while solutions with a pH greater than 7 are basic or alkaline solutions. This sensor gives us a signal related to the hydrogen ion concentration measured by a pH electrode. The isopotential point pH of 7.00 (0 mV), measured on a range from 0 to 14, has an accuracy lower than 15 mV. The relationship between the pH value and the output voltage is E = 59.16 (mV/pH). The pH sensor is connected to a wireless node sensor that provides the necessary electronics to control the different hardware parts through micro-pumps which take the different solutions and liquids from containers. 

**Figure 1:**
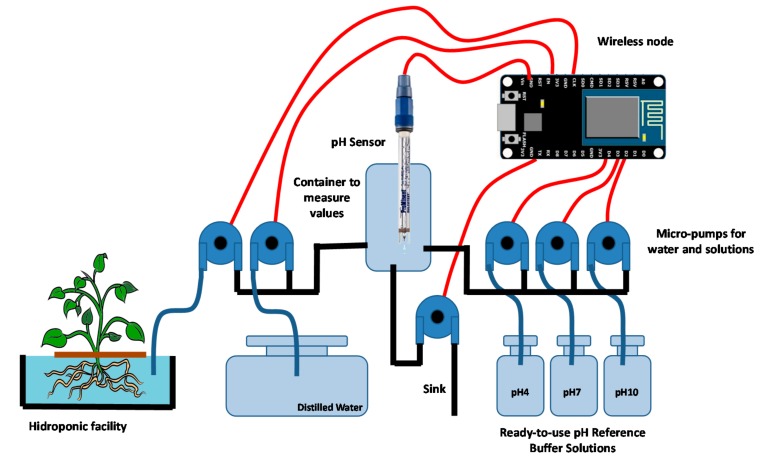
Auto-calibrated pH sensor and wireless node.

In order to obtain accurate measurements, the sensor needs to be periodically calibrated. To this end, three ready-to-use pH reference buffer solutions to perform decalibration and provide distilled water to clean the probe are required. Firstly, we need to clean the glass bulb of the electrode. Next, the pH sensor has to be turned to calibration mode. After injecting the calibration solution into a beaker, the electrode is immersed on it with a micro-pump. Figure 1 shows our auto-calibrated pH sensor with the micro-pumps that provide the measured values of required liquids to the container, to perform firstly, the calibration process and secondly, the measurement of the pH value of the nutrient solution in our hydroponic duct.

There are different parameters and factors that cause an increase in the pH value over time. For example, if the water contains wastewater, the pH can increase by 1 point in a week. This type of change occurs over a long period of time so it is easy to act on. On the other hand, bacteria and microorganisms change the CO_2_ levels in the water, so they can also affect the pH. If the presence of algae is significant, the pH will increase because the CO_2_ will be removed from the solution. Bacteria can transform certain forms of nitrogen, thus having the effect of increasing the acidity. Large amounts of CO_2_ in the air generate more CO_2_ in the nutrient solution and vice versa. For this type of problem, it may be advisable to measure the solution every 2 days. Finally, we must consider that in medium–high temperature conditions, when the pH of the nutrient solution is in the range of 7.5, the correct absorption of nutrients by the plant is affected.

### 3.2. System Operation

The auto-calibrated pH sensor is a precision agriculture system which is part of a WSN, and a smart framework software supports the control of irrigation process on greenhouses. This subsection is focused on the design of the algorithm.

The main function of the control algorithm is to detect the presence of carbonates or bicarbonates in the nutrient solution which determines the pH values. The bicarbonate ion (HCO_3_) is present at pH levels between 4.5 and 8.3, and the carbonate ion (CO_3_) is present in the nutrient solutions with pH levels above 8.3. The calcium carbonate salts have very low solubility so, once the precipitate has been produced, it is very difficult to restore the pH value of the water. The best way to prevent this problem is to carry out continuous treatment with commercial acids that neutralize and destroy the bicarbonates found in the nutrient solution. In this way, we can avoid their combination with calcium ions in the nutrient solution or with those that are added as nutrients. The amount of acid that needs to be incorporated into the nutrient solution depends on the volume of water that is to be treated and the bicarbonate content of the nutrient solution Excess acid will produce a drop in the pH level to a value that could be lethal to crops.

The use of laboratory analysis to control the nutrient solution could give bad results due to unexpected changes in the quality of the nutrient solution. For this reason, it is necessary to know the data values within the shortest possible time frame. To solve this issue, we developed an acid evaluation method and algorithm to obtain a neutralization curve to determine the bicarbonate content. In this method, the nodes periodically send data from a pH sensor to the middleware where the node connectivity is checked to verify the correct operation of the entire system. The nodes turn on the sensors to start the data capturing process. After that, a decision system based on the learning of association rules in conjunction with Drools starts to analyze data and determines the amount of acid that should be injected to obtain optimal water quality.

The auto-calibration model was designed with the objective of minimizing the amount of false data gathered and to offer a tool focused on disease prevention for managing greenhouse quality water in a sustainable way. The model is based on the following chemical reaction (See Equation (1)):

(1) $$H^{+}+{\mathrm{HCO}}_{3^{-}}↔{\mathrm{CO}}_{2}+H_{2}O$$

Table 1 represents the results of our previous experiments that describe the values of acid in nutrient solution samples registered in our greenhouse. The scenario being tested is a small tunnel-type greenhouse, 15 m long by 9 m wide. These data are stored on the MySQL Server database with references values of T^a^, pH and EC. The goal of our system is to neutralize bicarbonates to optimize the irrigation functionality. The density increases because the purer the acid is, the greater the amount of neutralization required.

This system was also conceived to help farmers to monitor the quality of nutrient solutions and to increase the quality of crops produced by presenting information about parameters and recommendations to support decision-making. Thus, the auto-calibrated pH sensor covers three basic needs:

1. It keeps a record of the main parameters that affect the development of irrigation and fertilization, through the ability to use smart core decision processes to identify deficiencies in water quality values, by using a wide range of devices connected in a WSN.
2. It assists in preventing crop diseases through predictive models by obtaining ad-hoc alerts from the farm. Specifically, the algorithm automates the detection of both primary and secondary quality water tests and sends alerts to the users. It is possible to use WSN as a greenhouse controller [30]. The algorithm is hosted on a central server that checks the status of the infection once a day using the environmental parameters collected from the nodes. Then, the auto-compensative index is calculated, in accordance with Table 1. When the accumulated index value exceeds a threshold (e.g., 80%), an alert is sent to the user.
3. It helps to reduce and prevent environmental pollution, reuse wastewater for irrigation and increase the auto-management of crop systems, in accordance with sustainable Common Agricultural Rural Development rules promoted by the European Union in Common Agricultural Policy.

Because the main function of our algorithm is to obtain a pH between 5.5 and 6, we must leave 0.5 ${\mathrm{meqL}}^{-1}$ of bicarbonate in the solution so that the pH stays within the desired range. The presence of 0.5 ${\mathrm{meqL}}^{-1}$ of bicarbonate does not produce precipitation of calcium carbonate in the irrigation network. This is the reference value used when determining the level of maintenance required in an irrigation system to reduce the risk of emitters being blinded due to calcium carbonate.

### 3.3. Network Architecture

**Figure 2:**
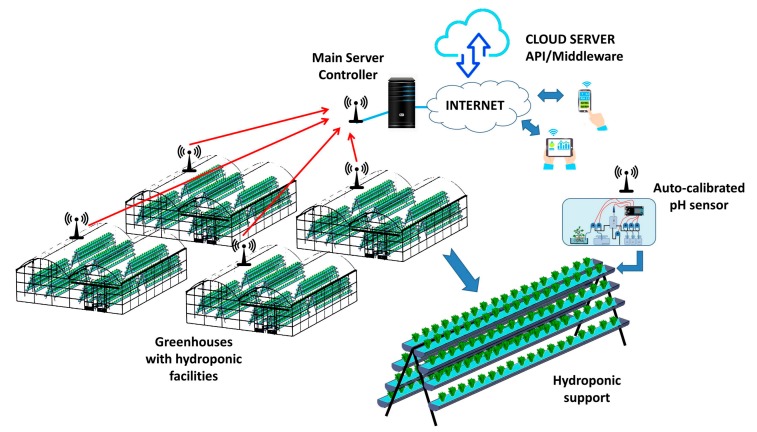
Network architecture.

As we mentioned before, the designed pH sensor is integrated into WSN architecture that monitors several greenhouses containing hydroponic systems. Manual interpretation is a time consuming and expensive task. For these reasons, we introduced a WSN for gathering data. Figure 2 shows the network scenario with several greenhouses. The different nodes of each greenhouse send the data from a pH sensor, T^a^ sensor and EC sensor to the server which is in charge of controlling the entire facility and is directly connected to the Internet. The data is stored in a DB placed in the cloud. The owners can access the stored data through personal devices in order to check historical gathered data and confirm the correct operation of different systems.

In this case, we selected the NRF24L01 transceiver from the Nordic Semiconductor Company. The communications chip chosen was an 8-bit Atmel which is characterized by its low power consumption. We have found some proposals and recommendations regarding the use of the NRF24L01 transceiver, but in all of them, we can only see the part of the data transmission from a node to its coordinator. In previous work with the NRF24L01 transceiver, the communications protocol pattern was based on sending the data from the node to the mesh network so that it eventually reached the coordinator. These networks have mainly been developed in WSNs to send data about variables. In our mesh network proposal, the routing method was based on the node identifiers, rather than using a prefixed routing table. Finally, the nodes were protected by IP66 boxes.

## 4. Middleware Design

The main goal of middleware software is to analyze historic data related to the acid density ratio and to modify the acidity values of the nutrient solution to ensure the correct growth of the crops. The DB contains the reference values shown in Table 1 to be applied in the algorithm. This section shows the algorithm in charge of controlling the node connection, the measurement process and the adequacy of water levels.

**Figure 3:**
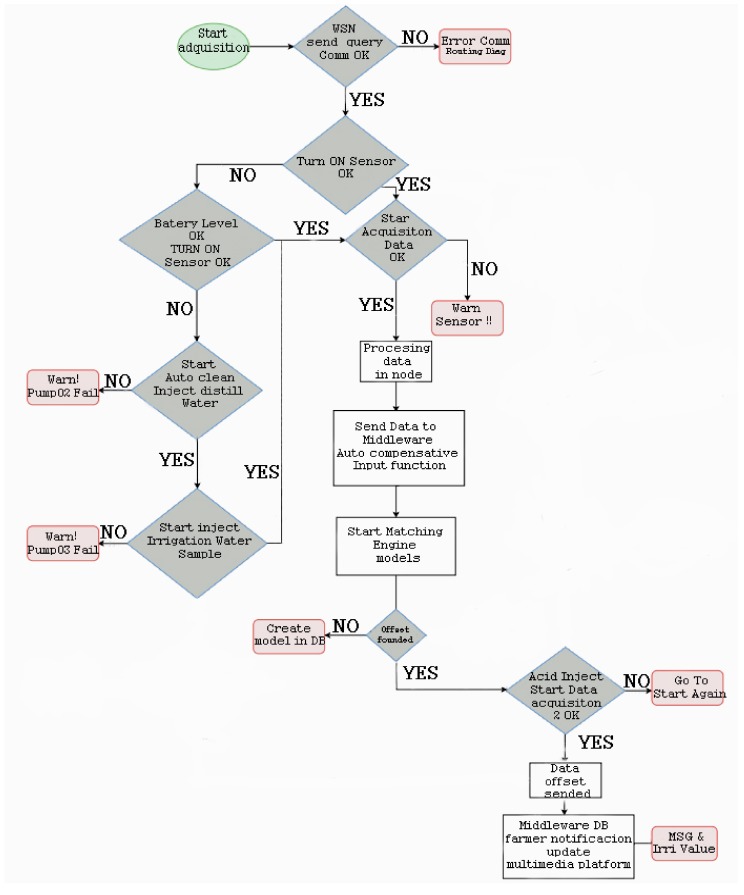
Flow diagram of our intelligent diagnosis algorithm.

Figure 3 shows the operation algorithm of the entire system. The decision algorithm in charge of analyzing bicarbonates parses the quality water model and tries to match the rules according to real-time sensor data and the historic data sensor stored in the DB. A diagnosis is achieved if all rules are successfully matched. Otherwise, the probability of occurrence is calculated if only some of the rules match. 

As Algorithm 1 shows, the system firstly checks the wireless connectivity and nodes’ status. In this phase, the system determines if there are any problems related to the battery level or any other hardware issues. If the values from the sensors are out of range, the system starts the auto-clean process and the calibration tasks. The next step is data gathering, and if these values are inside the normal range, the data is wirelessly sent to the middleware located in the server. When the middleware receives new data, it starts to compare the new values with the historic data and values of equivalent dosages preconfigured in the algorithm. After processing the data, the value of acid to be added to the nutrient solution is calculated. At this point, the acid micro-pumps are enabled to inject the amount of acid required. Finally, the system rechecks the pH level to verify that neutralization process has been successfully performed.

**Algorithm 1.** Pseudocode of Flow Diagram(0) WSN Conectivity OK; (1) Get data; (2) Start intelligent diagnostic Rule Engine; (3) Determine the current status of the pH, electrical conductivity (EC) and acidity parameters based on the rules and sensor data; (4) If the current state and the historical state are different, then   (5) The current status is regarded as the historical status, and the current time is recorded; (6) Otherwise   (7) Compute the state of the battery;      (8) If the duration is within the limit, then      (9) Intelligent diagnosis is delivered;     (10) Otherwise        (11) Auto-clean the sensor and inject new water sample        (12) Go to (2)      (13) End      (14) Otherwise      (15) Go to (1)     (16) End (17) End

The designed middleware/decision system is implemented in Java Drools and monitored by PHP and HTML5 languages, and the service is provided services through the Internet. Users can remotely login into the system via the Internet, browse and query all of the information, including historical sensor data and alert information, real-time sensor data and alert information, expert knowledge, intelligent diagnoses and other services. Regarding the storage of the collected data, a MySQL database was selected, since it is widely used and there is a strong user community behind it. Moreover, the back-end and front-end servers chosen were compatible with such a database.

## 5. Results

This section shows the results of the developed system, focusing on three important aspects: the quality of the collected data, the communication network and the power consumption of nodes.

The system was used in tomato crops and lettuce crops placed in the same small greenhouse (15 m length × 9 m wide × 3 m high, tunnel type). The choice of these crops was because they are some of the most suitable for cultivation with this technique [31].

### 5.1. Auto-Calibrated pH Sensor Algorithm Performance

According to the values shown in Table 1, if a 56% nitric acid solution is required, we will obtain nutrient solution of 1.35 kg/dm^3^ of density. This means that in order to provide a ${\mathrm{meqL}}^{-1}$ of nitric acid which weighs 63 mg (of 56% of wealth), we have to add 112.5 mg (63/56) of acid. Therefore, if we have a water density of 1.35, we should add 84 microliters (112.5/135) of 56% nitric acid to obtain a ${\mathrm{meqL}}^{-1}$ of pure nitric acid. 

In order to see how the pH sensor is able to neutralize a dose of water by providing the volume of required acid to neutralize the sample, we progressively added 5 ${\mathrm{meqL}}^{-1}$ of bicarbonate to a water sample with an initial pH of 8. Additionally, we took into account that it is necessary to have samples of water doses and acid very accurate so that the carbonates are correctly neutralized, with 0.5 ${\mathrm{meqL}}^{-1}$ of bicarbonate left unneutralized. After the experiment, the final sample will have 420 μL of nitric acid with a purity of 56%.

**Figure 4:**
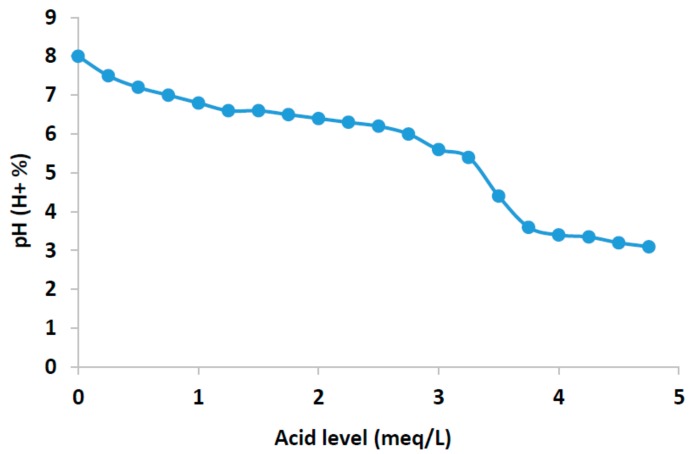
pH of water.

When the value of acidity changes, there is an ion combination that generates important changes in water. In concrete, the increase of carbonate in water generates changes in water temperature, water conductivity and the presence of H^+^. Figure 4 shows the pH values of water solution as a function of the ${\mathrm{meqL}}^{-1}$ of the added acid. The pH of the nutrient solution is an issue to take into account, but it must be considered in the context of the alkalinity of the water. It is a common misconception that the pH of the water will determine the pH of the growth substrate. In most cases, the alkalinity (buffering capacity) of the nutrient solution has a greater effect on the substrate pH than the pH of the water. However, high pH can be indicative of high alkalinity, so if a nutrient solution has a pH greater than 7, the alkalinity should be tested. High pH in conjunction with high alkalinity can cause serious issues for crops in soilless growing systems.

Starting from the initial pH values of the water sample, after adding the first doses of acid, in the resulting solution, most of the protons initially react with the carbonates in the water, but some of them remain free in the water. The results are shown in graphs in accordance with the reference table for acid dosages in relation to pH. This causes a decrease in pH.

**Figure 5:**
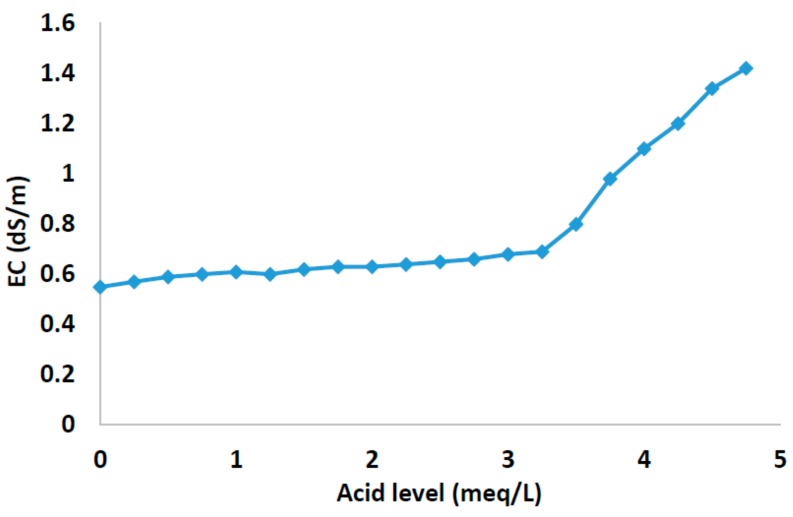
EC of water.

Figure 5 shows the EC of water as a function of the ${\mathrm{meqL}}^{-1}$ of added acid. As we can see, the EC increases by 2–3% when a new ${\mathrm{meqL}}^{-1}$ acid is added (from 2.8 ${\mathrm{meqL}}^{-1}$). Many commercial EC meters automatically normalize the readings at 25 °C. Our algorithm determines the inflection point as a result of two stretch intersections of bicarbonates. Soluble salts are detected by measuring the EC of the nutrient solution since it is the dissolved ions that conduct the electrical current through the water. Pure, distilled water does not conduct electricity. EC is usually reported as a unit of electrical resistance, the scientific standard for measuring EC. The metric is equivalent to the mho ($\Omega^{-1})$. The level of soluble salts that is acceptable in a nutrient solution varies from near zero for hydroponic systems to up to about 1 mS/cm for potted crops. A higher conductivity means a greater quantity of salts in the water which can lead to plant injury under certain conditions. Through this graph, we can see that the relationship between the amount of acid injected and the EC value based on the real scenario tested determines the quantity of acid that has to be injected to neutralize bicarbonates. It is shown as the second stretch increase.

**Figure 6:**
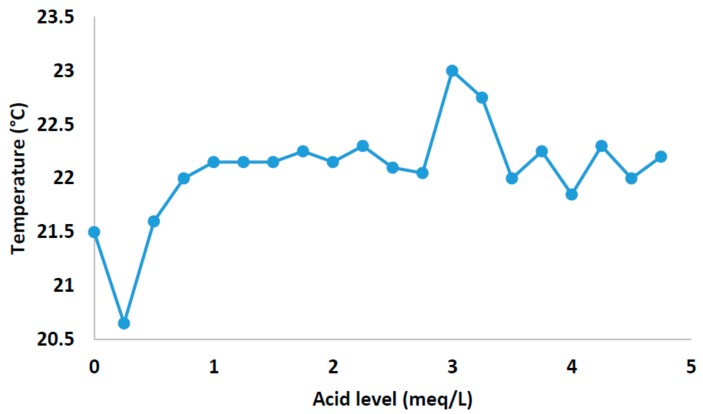
Data collected on node 3.

Figure 6 shows the relationship between the temperature and the ${\mathrm{meqL}}^{-1}$ of added acid. As we can see, in the first stretch of the graph, there a negative slope; this is due to the ionic equivalent present in the water. When the process of auto-compensation is carried out and the acid is injected (from 2.8 ${\mathrm{meqL}}^{-1}$), the bicarbonate ion is neutralized and the second part of the graph fluctuates more than the first part.

**Figure 7:**
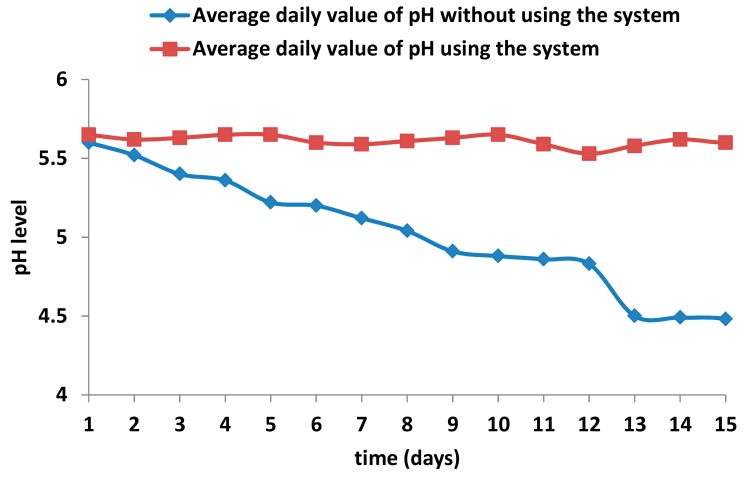
Evolution of pH values as a function of the time.

Finally, we test two possible cases, i.e., the evolution of the pH level when the auto-calibrated pH sensors and our algorithm are not used and when the designed system is used (see Figure 7). The experiment was carried out over two weeks, in which a measure was taken every 6 hours to calculate the average daily pH value. This comparison intends to show the differences between a system that uses an unmanned auto-calibration system and when sensors without assistance are used. When we do not carry out rectifications of the nutrient solution, the pH value of the water contained in the gutters loses the desirable value for the crops as time goes on, and to weather conditions such as rain or water evaporation due to excess heat. However, when using our system, the acidity level of the irrigation water is modified according to the water of the gutters, to maintain the overall desirable pH level of the water offered to the crops. As we can see, when the system is used, the pH value is stable at values between 5.5 and 5.6, while in a scenario where our system is not used, the value of pH decreases as a function of the time reaching values too low for crops. A pH lower than 5.5 is a potential problem, and the elemental constituents of the water should be examined. Some factors than can generate these changes are the rain or water evaporation in gutters due excess of heat.

### 5.2. Wireless Sensor Network Performance

**Figure 8:**
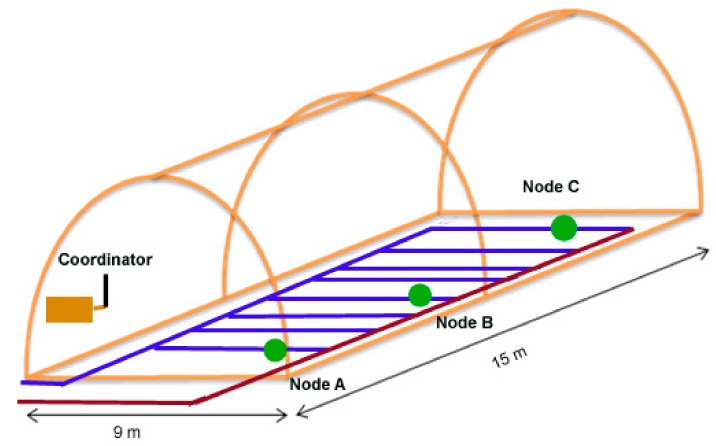
Experimental network setup in greenhouse.

In order to determine the network’s performance, we have deployed the network shown in Figure 8. Node A was placed 7.50 m away from coordinator, located in the main wall of the greenhouse. It was installed at a height of 60 cm and the distance to the edge of the dense tomato foliage was 40 cm. Node B had a distance of 11.8 m to the coordinator node, and it was placed at the height of 60 cm. The length between the first plants and the device was 74 cm. Node C was at the end of greenhouse (at 14.5 m from main wall). The distance from the node to the edge of the foliage was 35 cm. Periodical sleep and wake modes were applied to reduce energy consumption. Each node woke up and turned on its radio for 15 s and went then back to sleep and turned off its radio for 255 s (4 min 15 s). For each slot time, only one of the three nodes equipped with sensors read data from the sensors and waited for the data request from the coordinator. The coordinator was in charge of taking care of the data received; the rest of the nodes were only able to reply to the coordinator’s requests. Thus, the coordinator acted as the master device which polled data from the sensor nodes at certain time periods.

Due to climate change or the presence of vegetation between the nodes and coordinator node, it is possible for errors to occur. After capturing the network traffic during 70 min, we detected a very low error rate of 1.6%.

The radio system supports an automatic confirmation function (autoACK), activated by default, in which the receiving radio changes to transmission mode after reception and recognizes the reception of data. This helps to maintain a bidirectional channel.

In the first test, a simple scenario is created with three nodes, A, B and C, with the following exchange of messages:

1. A sends to B
2. A sends to C
3. B answers A
4. A already knows that B has the right message.
5. C answers A
6. One already knows that C has the right message.

**Figure 9:**
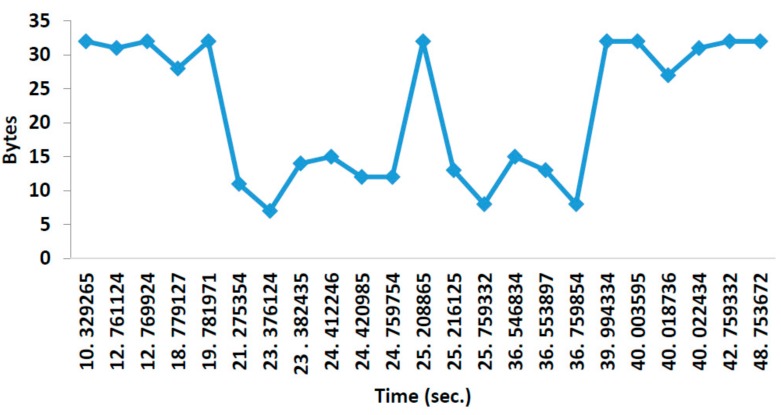
Network traffic, point to point.

Figure 9 shows the consumption in bytes of the network for the message exchange explained. According to the recommendations of the manufacturers, if we keep the automatic confirmation active, we are limited to only five nodes in the network. Therefore, for this work, it is necessary to deactivate this function and to expand a multi-network with more than six nodes. This function was implemented by software. Our implementation allowed the use of up to 255 addresses. The frame format consists of the origin address (first byte), the destination address (second byte), forward address (third byte), acknowledgement (forth byte) and data (from fifth to 32nd byte). So, our frame has a length of 32 bytes.

### 5.3. Radio Node Energy Consumption

**Figure 10:**
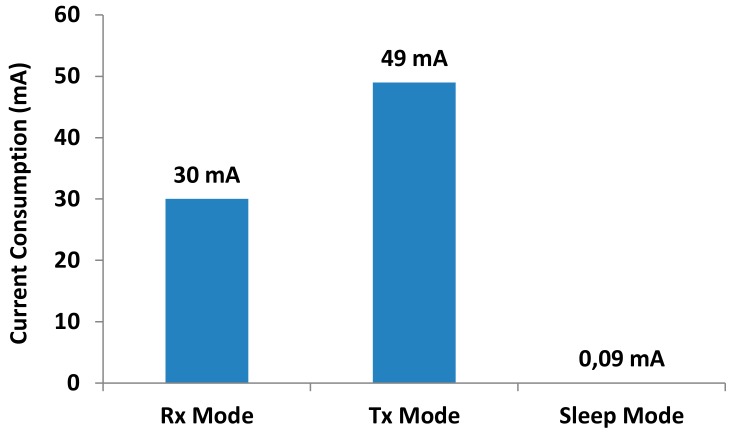
Radio node current consumption.

Finally, Figure 10 shows the current consumption values of these radio communications modules when they are operating at 3.3 V. When the node is transmitting, the current value is around 30 mA. The current consumption of the node when transmitting data increases up to 49 mA. Finally, when the node is in sleep mode, the current consumption is radically reduced to 90 μA.

The benefits of the automation of the process of taking measures are the reduction in the maintenance costs of the facilities and the improvement in the analysis process which with the classic manual analysis methods, can support 20 min of processing per sample. Acid data acquisition should be done every 45–50 min during watering. In this way, we can ensure the quality of the nutrient solution.

## 6. Conclusions

This paper has presented a smart auto-calibrated pH sensor allowing the data gathering and adjustment of the quality of nutrient solutions to optimize watering in precision agriculture. Specifically, our smart auto-calibrated system supports decision-making to control false data acquisition. The sensor is controlled by a wireless node that is part of a WSN which is controlled by decision-making middleware [32]. Our system notifies the farmer when values have reached a threshold to apply corrective measures. Thus, the system helps to avoid the excessive use of acid and nutrient solution, reducing the impact on the environment.

The information collected by our WSN is presented through a user-friendly web portal for management and visualization. The web-based system can be accessed from any computer, tablet or smartphone with the only requirement being having a browser and an Internet connection. To sum up, all results obtained confirm that smart middleware is a good tool for auto-control of data and remote management of greenhouses to automate the detection of blind drippers and water quality.

After testing and analyzing the results, we conclude that the use of our system can help farmers to better control the process of watering. One of the main benefits for farmers is that this system will reduce the worktime required for cleaning and calibrating sensors in large areas dedicated to indoor horticulture production and will enhance the autonomy of greenhouses. Vertical agriculture could reduce the impact of the effects on the environment [12]. Our solution could be integrated to optimize input costs, allowing the restoration of thousands of hectares currently damaged by agriculture. Furthermore, this kind of detection not only would generate savings for the farmer, but also would reduce water consumption making the current horticultural production more sustainable.

The proposed system allows data gathering for decision support in farming. It helps users to easily make decisions in greenhouse production by providing descriptive, predictive and prescriptive analytics.

For future work, we will focus our efforts on providing more precise definitions of other parameters that can also affect water quality irrigation with the purpose of adding them to our real-time monitoring systems. We also want to check the integration of new rules into algorithms in autonomous irrigation controllers without human interaction. We will carry out a study to evaluate if our developed smart sensor could be a solution for basic commercial systems on the market.

## Figures and Tables

**Table 1:** Values of acid density and purity of the two acid types in our model.

Acid Density (kg/dm^3^)	% Purity Phosphorous Acid	% Purity Nitric Acid
1	16	15
1.1	18	18
1.15	26	24
1.2	34	33
1.25	40	40
1.3	46	48
1.35	53	56
1.4	57	65
1.45	63	77
1.5	68	95
1.55	73	100
